# Supplementary material for: Characterization of Streptomyces sporangiiformans sp. nov., a Novel Soil Actinomycete with Antibacterial Activity against Ralstonia solanacearum
Source: Microorganisms. 2019 Sep 17;7(9):360. doi: 10.3390/microorganisms7090360 (PMC6780108; doi:10.3390/microorganisms7090360)
Supplement: Supplementary file 1 [file microorganisms-07-00360-s001.pdf]

Fig. S1. Polar lipids composition of strain NEAU-SSA 1^{T}.

Abbreviations: DPG, diphosphatidylglycerol; PE, phosphatidylethanolamine; OH-PE, hydroxy-phosphatidylethanolamine; PI, phosphatidylinositol; PIM, phosphatidylinositol mannoside; PL, unidentified phospholipid. a, using ethanolic phosphomolybdic acid hydrate reagent. b, using ninhydrin reagent; c, using molybdenum blue reagent; d, using anisaldehyde reagent.

1st dimension: Chloroform : Methanol : Water (65 : 25 : 4, v/v);

2nd dimension: Chloroform : Acetic acid : Methanol : Water (80 : 18 : 12 : 5, v/v).

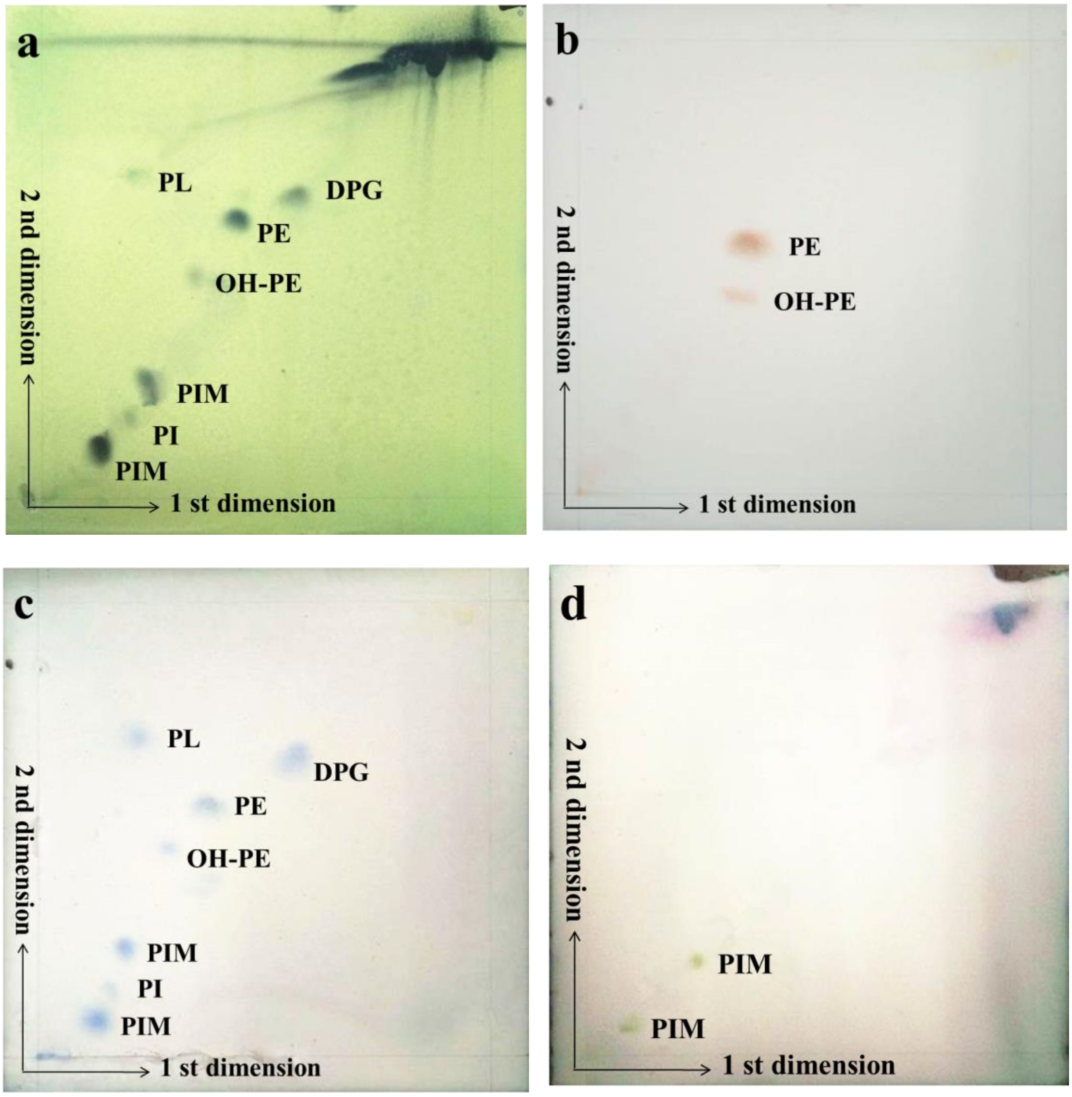

Fig. S1

Fig. S2. Maximum-likelihood tree showing the phylogenetic position of strain NEAU-SSA 1^T (1412 bp) and the related species based on 16S rRNA gene sequences. The out-group used was *Kitasatospora setae* LM-6054^T. Only bootstrap values above 50 % (percentages of 1000 replications) are indicated. Bar, 0.01 nucleotide substitutions per site.

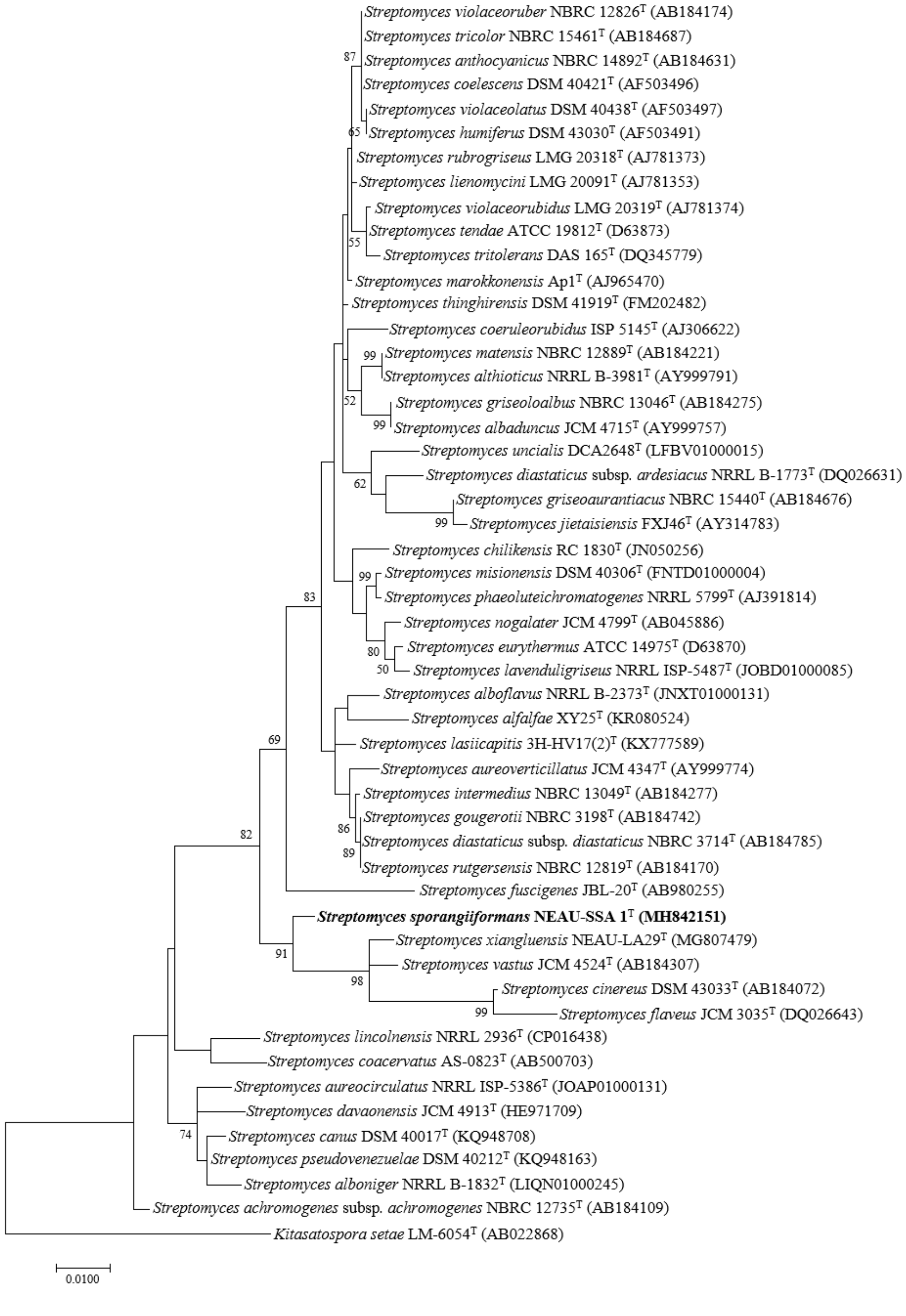

Fig. S2

Fig. S3. Maximum-likelihood tree based on MLSA analysis of the concatenated partial sequences from five housekeeping genes (*atp*D, *gyr*B, *rec*A, *rpo*B, and *trp*B) of isolate NEAU-SSA 1^T (in bold) and related taxa. Only bootstrap values above 50 % (percentages of 1000 replications) are indicated. *Kitasatospora setae* LM-6054^T was used as an out-group. Bar, 0.05 nucleotide substitutions per site.

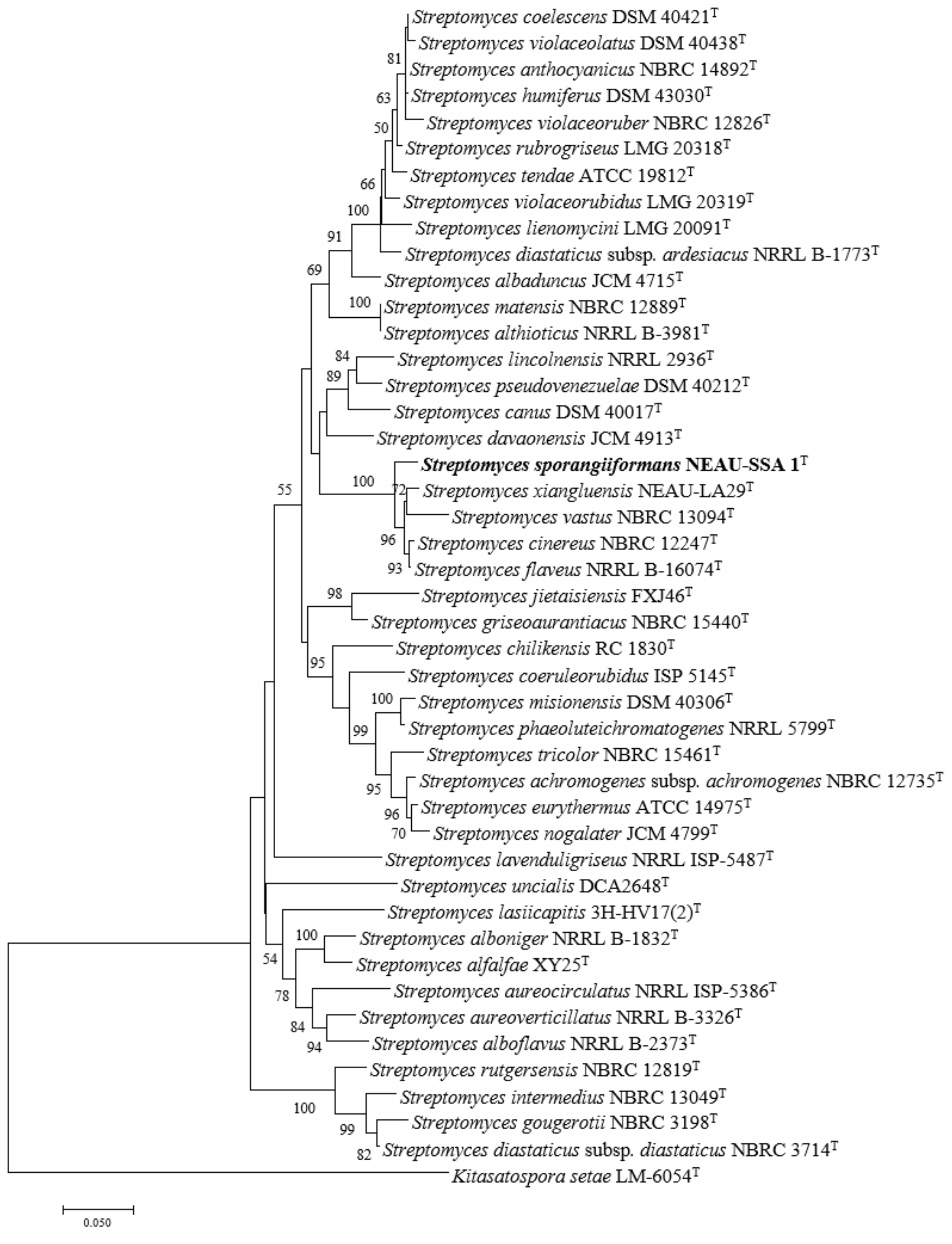

Fig. S3

Table S1. Growth and cultural characteristics of strain NEAU-SSA 1^T

Agar Media	Growth	Aerial mycelium colour	Colony colour	Substrate mycelium colour
ISP 1	Moderate	Greenish White	Deep yellow	Deep yellow
ISP 2	Moderate	Greenish White	Pale Yellowish Green	Pale Yellowish Green
ISP 3	Good	Yellowish White	Pale yellow	Pale yellow
ISP 4	Good	Yellowish White	White	White
ISP 5	Moderate	Yellowish White	White	White
ISP 6	Moderate	Yellowish White	Light yellow	Light yellow
ISP 7	Good	Yellowish Gray	Dark yellow	Dark yellow
Czapek's	Moderate	Yellowish White	White	White
Nutrient	Good	Greenish White	Moderate Greenish Yellow	Moderate Greenish Yellow
Bennett's	Poor	Yellowish White	Moderate Greenish Yellow	Moderate Greenish Yellow

Table S2. MLAS distance values for selected strains in this study.

Strains: 1, NEAU-SSA 1^T; 2, *Streptomyces xiangluensis* NEAU-LA29^T; 3, *Streptomyces aureoverticillatus* JCM 4347^T; 4, *Streptomyces gougerotii* NBRC 3198^T; 5, *Streptomyces diastaticus* subsp. *diastaticus* NBRC 3714^T; 6, *Streptomyces rutgersensis* NBRC 12819^T; 7, *Streptomyces lasiicapitis* 3H-HV17(2)^T; 8, *Streptomyces rubrogriseus* LMG 20318^T; 9, *Streptomyces intermedius* NBRC 13049^T; 10, *Streptomyces lincolnensis* NRRL 2936^T; 11, *Streptomyces coelescens* DSM 40421^T; 12, *Streptomyces anthocyanicus* NBRC 14892^T; 13, *Streptomyces vastus* JCM 4524^T; 14, *Streptomyces diastaticus* subsp. *ardesiacus* NRRL B-1773^T; 15, *Streptomyces violaceoruber* NBRC 12826^T; 16, *Streptomyces tricolor* NBRC 15461^T; 17, *Streptomyces violaceolatus* DSM 40438^T; 18, *Streptomyces humiferus* DSM 43030^T; 19, *Streptomyces eurythermus* ATCC 14975^T; 20, *Streptomyces lienomycini* LMG 20091^T; 21, *Streptomyces misionensis* DSM 40306^T; 22, *Streptomyces matensis* NBRC 12889^T; 23, *Streptomyces pseudovenezuelae* DSM 40212^T; 24, *Streptomyces tendae* ATCC 19812^T; 25, *Streptomyces albaduncus* JCM 4715^T; 26, *Streptomyces althioticus* NRRL B-3981^T; 27, *Streptomyces cinereus* DSM 43033^T; 28, *Streptomyces jietaisiensis* FXJ46^T; 29, *Streptomyces aureocirculatus* NRRL ISP-5386^T; 30, *Streptomyces canus* DSM 40017^T; 31, *Streptomyces nogalater* JCM 4799^T; 32, *Streptomyces phaeoluteichromatogenes* NRRL 5799^T; 33, *Streptomyces flaveus* JCM 3035^T; 34, *Streptomyces coeruleorubidus* ISP 5145^T; 35, *Streptomyces lavenduligriseus* NRRL ISP-5487^T; 36, *Streptomyces alboniger* NRRL B-1832^T; 37, *Streptomyces achromogenes* subsp. *achromogenes* NBRC 12735^T; 38, *Streptomyces alboflavus* NRRL B-2373^T; 39, *Streptomyces alfalfae* XY25^T; 40, *Streptomyces chilikensis* RC 1830^T; 41, *Streptomyces davaonensis* JCM 4913^T; 42, *Streptomyces griseoaurantiacus* NBRC 15440^T; 43, *Streptomyces uncialis* DCA2648^T; 44, *Streptomyces violaceorubidus* LMG 20319^T; 45, *Kitasatospora setae* LM-6054^T

Strain	MLSA (Kimura 2-parameter) distance																																											
	1	2	3	4	5	6	7	8	9	10	11	12	13	14	15	16	17	18	19	20	21	22	23	24	25	26	27	28	29	30	31	32	33	34	35	36	37	38	39	40	41	42	43	44	45
1	-																																												
2	0.029	-																																											
3	0.096	0.096	-																																										
4	0.105	0.112	0.115	-																																									
5	0.107	0.113	0.110	0.021	-																																								
6	0.120	0.125	0.105	0.062	0.042	-																																							
7	0.092	0.095	0.081	0.119	0.113	0.116	-																																						
8	0.093	0.096	0.102	0.099	0.102	0.097	0.104	-																																					
9	0.113	0.119	0.113	0.040	0.025	0.051	0.115	0.099	-																																				
10	0.075	0.075	0.104	0.105	0.105	0.115	0.104	0.075	0.116	-																																			
11	0.090	0.092	0.098	0.095	0.097	0.096	0.103	0.009	0.098	0.080	-																																		
12	0.090	0.092	0.096	0.095	0.097	0.096	0.101	0.008	0.098	0.078	0.002	-																																	
13	0.044	0.033	0.114	0.128	0.129	0.142	0.112	0.114	0.135	0.089	0.112	0.112	-																																
14	0.095	0.095	0.095	0.096	0.096	0.088	0.096	0.023	0.100	0.081	0.026	0.025	0.115	-																															
15	0.093	0.094	0.099	0.094	0.096	0.096	0.105	0.018	0.094	0.088	0.010	0.010	0.112	0.035	-																														
16	0.093	0.099	0.108	0.107	0.095	0.098	0.106	0.095	0.097	0.087	0.093	0.093	0.114	0.089	0.095	-																													
17	0.094	0.093	0.098	0.098	0.101	0.100	0.106	0.013	0.102	0.082	0.004	0.005	0.115	0.030	0.012	0.095	-																												
18	0.091	0.091	0.097	0.095	0.097	0.096	0.102	0.009	0.098	0.078	0.002	0.001	0.111	0.025	0.011	0.093	0.006	-																											
19	0.092	0.092	0.102	0.105	0.096	0.095	0.097	0.081	0.098	0.086	0.080	0.080	0.110	0.077	0.089	0.029	0.084	0.081	-																										
20	0.095	0.094	0.103	0.101	0.102	0.103	0.104	0.026	0.104	0.084	0.023	0.021	0.116	0.030	0.029	0.089	0.019	0.022	0.085	-																									
21	0.099	0.104	0.104	0.096	0.088	0.090	0.101	0.081	0.091	0.089	0.081	0.079	0.123	0.078	0.083	0.045	0.083	0.079	0.042	0.081	-																								
22	0.088	0.089	0.092	0.112	0.111	0.103	0.103	0.069	0.113	0.078	0.064	0.064	0.107	0.060	0.070	0.081	0.067	0.065	0.079	0.061	0.088	-																							
23	0.072	0.069	0.099	0.104	0.103	0.111	0.097	0.063	0.110	0.037	0.068	0.066	0.086	0.070	0.075	0.079	0.070	0.066	0.077	0.075	0.087	0.068	-																						
24	0.095	0.095	0.104	0.096	0.099	0.095	0.104	0.013	0.102	0.075	0.018	0.017	0.116	0.025	0.027	0.091	0.021	0.017	0.081	0.025	0.079	0.067	0.065	-																					
25	0.088	0.085	0.096	0.102	0.098	0.101	0.102	0.043	0.103	0.073	0.045	0.043	0.103	0.043	0.051	0.081	0.046	0.043	0.075	0.048	0.086	0.052	0.067	0.043	-																				
26	0.088	0.089	0.092	0.112	0.111	0.103	0.103	0.069	0.113	0.078	0.064	0.064	0.107	0.060	0.070	0.081	0.067	0.065	0.079	0.061	0.088	-0.000	0.068	0.067	0.052	-																			
27	0.026	0.016	0.095	0.108	0.108	0.120	0.088	0.089	0.113	0.071	0.087	0.087	0.032	0.088	0.089	0.095	0.090	0.086	0.090	0.091	0.100	0.087	0.063	0.091	0.078	0.087	-																		
28	0.103	0.106	0.102	0.116	0.119	0.121	0.114	0.099	0.123	0.096	0.096	0.094	0.120	0.097	0.102	0.092	0.099	0.094	0.085	0.095	0.090	0.083	0.091	0.099	0.095	0.083	0.100	-																	
29	0.102	0.104	0.063	0.116	0.111	0.110	0.104	0.105	0.120	0.091	0.105	0.103	0.122	0.111	0.105	0.108	0.106	0.104	0.107	0.109	0.105	0.098	0.089	0.108	0.101	0.098	0.098	0.098	-																
30	0.081	0.081	0.097	0.112	0.111	0.110	0.097	0.066	0.111	0.045	0.068	0.066	0.098	0.071	0.077	0.088	0.072	0.066	0.081	0.075	0.090	0.063	0.045	0.070	0.066	0.063	0.073	0.083	0.093	-															
31	0.092	0.094	0.106	0.111	0.100	0.104	0.100	0.091	0.103	0.088	0.088	0.088	0.109	0.082	0.095	0.038	0.092	0.088	0.016	0.091	0.051	0.078	0.082	0.090	0.075	0.078	0.089	0.088	0.109	0.083	-														
32	0.097	0.100	0.101	0.092	0.081	0.082	0.098	0.075	0.088	0.082	0.075	0.074	0.120	0.073	0.078	0.042	0.078	0.074	0.038	0.077	0.011	0.082	0.081	0.075	0.075	0.082	0.098	0.093	0.098	0.085	0.047	-													
33	0.025	0.013	0.095	0.106	0.107	0.119	0.090	0.088	0.113	0.071	0.084	0.084	0.030	0.087	0.087	0.093	0.088	0.083	0.087	0.090	0.097	0.085	0.063	0.090	0.081	0.085	0.005	0.097	0.098	0.075	0.088	0.096	-												
34	0.092	0.093	0.102	0.098	0.104	0.108	0.100	0.080	0.112	0.078	0.080	0.078	0.112	0.076	0.087	0.066	0.082	0.079	0.060	0.080	0.061	0.077	0.079	0.077	0.072	0.077	0.091	0.090	0.103	0.084	0.071	0.053	0.088	-											
35	0.114	0.113	0.092	0.116	0.120	0.115	0.114	0.104	0.115	0.108	0.105	0.104	0.134	0.101	0.096	0.112	0.107	0.104	0.107	0.105	0.106	0.091	0.096	0.108	0.099	0.091	0.107	0.091	0.099	0.098	0.106	0.103	0.107	0.103	-										
36	0.098	0.096	0.065	0.100	0.100	0.094	0.091	0.094	0.102	0.096	0.093	0.091	0.117	0.094	0.091	0.103	0.096	0.092	0.094	0.096	0.094	0.090	0.094	0.096	0.095	0.090	0.095	0.104	0.074	0.088	0.097	0.091	0.093	0.088	0.096	-									
37	0.088	0.090	0.104	0.104	0.095	0.095	0.102	0.081	0.098	0.080	0.079	0.079	0.180	0.078	0.087	0.033	0.083	0.079	0.013	0.084	0.041	0.077	0.071	0.082	0.071	0.077	0.088	0.084	0.106	0.078	0.019	0.038	0.085	0.061	0.104	0.093	-								
38	0.095	0.095	0.042	0.107	0.107	0.106	0.075	0.096	0.106	0.100	0.095	0.093	0.113	0.091	0.090	0.099	0.098	0.094	0.096	0.092	0.100	0.083	0.096	0.097	0.086	0.083	0.092	0.100	0.073	0.094	0.100	0.096	0.092	0.097	0.081	0.065	0.097	-							
39	0.095	0.093	0.064	0.101	0.095	0.081	0.081	0.096	0.100	0.094	0.095	0.093	0.113	0.092	0.092	0.100	0.096	0.093	0.095	0.098	0.084	0.087	0.087	0.092	0.096	0.087	0.091	0.106	0.075	0.094	0.097	0.081	0.089	0.088	0.099	0.036	0.094	0.067	-						
40	0.084	0.084	0.106	0.104	0.104	0.105	0.102	0.093	0.104	0.087	0.091	0.089	0.104	0.089	0.096	0.077	0.096	0.089	0.067	0.092	0.063	0.081	0.081	0.089	0.088	0.081	0.083	0.097	0.107	0.079	0.069	0.059	0.080	0.064	0.100	0.090	0.063	0.090	0.088	-					
41	0.076	0.082	0.092	0.103	0.104	0.098	0.094	0.063	0.115	0.060	0.067	0.065	0.101	0.061	0.073	0.085	0.071	0.066	0.074	0.070	0.080	0.065	0.051	0.068	0.062	0.065	0.074	0.096	0.098	0.061	0.080	0.076	0.074	0.068	0.091	0.087	0.070	0.087	0.086	0.076	-				
42	0.092	0.093	0.090	0.105	0.108	0.111	0.099	0.069	0.110	0.077	0.070	0.069	0.107	0.067	0.076	0.076	0.074	0.069	0.070	0.064	0.068	0.065	0.069	0.068	0.074	0.065	0.089	0.047	0.100	0.063	0.071	0.070	0.086	0.076	0.087	0.094	0.069	0.085	0.096	0.074	0.072	-			
43	0.124	0.124	0.107	0.120	0.121	0.115	0.118	0.115	0.114	0.113	0.114	0.114	0.138	0.113	0.117	0.115	0.117	0.114	0.115	0.115	0.104	0.104	0.109	0.116	0.114	0.104	0.121	0.113	0.117	0.098	0.121	0.106	0.121	0.115	0.113	0.091	0.114	0.108	0.095	0.124	0.107	0.105	-		
44	0.090	0.091	0.101	0.093	0.096	0.096	0.102	0.017	0.098	0.071	0.021	0.020	0.109	0.022	0.030	0.090	0.025	0.021	0.081	0.027	0.080	0.065	0.063	0.019	0.043	0.065	0.087	0.092	0.103	0.067	0.088	0.075	0.086	0.075	0.100	0.088	0.080	0.090	0.096	0.092	0.060	0.063	0.110	-	
45	0.225	0.217	0.215	0.193	0.194	0.203	0.201	0.191	0.199	0.205	0.192	0.190	0.230	0.197	0.194	0.224	0.193	0.190	0.212	0.199	0.208	0.205	0.201	0.196	0.199	0.205	0.215	0.204	0.221	0.210	0.210	0.206	0.216	0.213	0.199	0.197	0.203	0.206	0.199	0.199	0.208	0.205	0.230	0.188	-

Table S3 General features of the genome sequence of the type strain NEAU-SSA 1^T.

Strain	NEAU-SSA 1^T
Genome size (bp)	10364704
DNA GC content (%)	69.9
Contigs	352
N50	59982
Genome coverage	200x
Accession No.	VCHX00000000
Assembly method	SOAPdenovo v. 2.04 OR JUL-2018
Bioproject	PRJNA543950
Sequencing Technology	Illumina HiSeq
